# Anti-inflammatory function of apolipoprotein B-depleted plasma is impaired in non-alcoholic fatty liver disease

**DOI:** 10.1371/journal.pone.0266227

**Published:** 2022-04-12

**Authors:** Negar Sarmadi, Hossein Poustchi, Fatemeh Ali Yari, Amir Reza Radmard, Sara Karami, Abbas Pakdel, Parisa Shabani, Ali Khaleghian

**Affiliations:** 1 Department of Biochemistry, School of Medicine, Semnan University of Medical Sciences, Semnan, Iran; 2 Liver and Pancreatobiliary Diseases Research Center, Digestive Diseases Research Institute, Tehran University of Medical Sciences, Tehran, Iran; 3 Department of Radiology, Shariati Hospital, Tehran University of Medical Sciences, Tehran, Iran; 4 Department of Integrative Medical Sciences, Northeast Ohio Medical University, Rootstown, OH, United States of America; Kaohsiung Medical University Chung Ho Memorial Hospital, TAIWAN

## Abstract

**Background:**

Non-alcoholic fatty liver disease (NAFLD) is associated with an increased risk of cardiovascular events. HDL exerts various protective functions on the cardiovascular system including anti-inflammatory activity by suppressing adhesion molecules expression in inflammation-induced endothelial cells. This study was designed to search if the anti-inflammatory capacity of apolipoprotein B-depleted plasma (apoB-depleted plasma) is altered in NAFLD patients.

**Methods:**

A total of 83 subjects including 42 NAFLD and 41 control subjects were included in this cross-sectional study. Anti-inflammatory function of HDL was determined as the ability of apoB-depleted plasma to inhibit tumor necrosis factor-α (TNF-α)-induced expression of adhesion molecules in human umbilical vein endothelial cells (HUVECs).

**Results:**

Incubation of inflammation-stimulated HUVECs with the NAFLD patients’ apo-B depleted plasma led to higher levels of expression of adhesion molecules compared to the control subjects’ plasma samples, reflecting an impaired anti-inflammatory capacity of apoB-depleted plasma in the NAFLD patients. Impaired anti-inflammatory capacity of apoB-depleted plasma was correlated with fatty liver and obesity indices. After adjustment with obesity indices, the association of anti-inflammatory capacity of apoB-depleted plasma with NAFLD remained significant.

**Conclusion:**

Impaired anti-inflammatory activity of apoB-depleted plasma was independently associated with NAFLD.

## Introduction

With the rising prevalence of obesity and metabolic syndrome, NAFLD has become the most common chronic liver disease worldwide. Beyond liver-related morbidity and mortality, cardiovascular diseases remain to be the main cause of death in NAFLD patients [1]. There are several shared risk factors between NAFLD and CVD. Among them, a low HDL-C level has been suggested to play a pivotal role in the elevated risk of CVD in NAFLD patients [2]. However, there is not a clear-cut inverse relationship between low levels of HDL-C and risk of CVD [3]. Pharmacological strategies to increase HDL-C were not successful in decreasing CVD risk [4]. Besides, a high level of HDL-C has been reported in patients with CVD events [5, 6]. These clinical observations led to emerging the appreciation that the functionality of HDL particles might be a more accurate indicator for the risk of developing atherosclerosis [7].

HDL has various atheroprotective properties [3]. One of the important atheroprotective functions of HDL particles is suppressing the expression of adhesion molecules in endothelial cells upon exposure to proinflammatory stimuli, namely anti-inflammatory function [3]. Previous studies unraveled the impaired HDL anti-inflammatory functionality in cardiovascular and other metabolic disorders. A study on non-MI subjects, non-ST segment elevation MI patients (STEMI), and STEMI patients showed the impaired anti-inflammatory function of HDL in STEMI patients compared to non-MI and non-STEMI subjects [3]. Another group also demonstrated severe impaired HDL anti- inflammatory capacity in MI patients [8]. Moreover, it has been shown that the HDL anti-inflammatory function was strongly impaired in type 2 diabetes patients [9, 10]. Previous studies showed an impaired HDL cholesterol efflux capacity in NAFLD patients [11, 12]. However, there is limited evidence indicating the possible alteration of HDL anti-inflammatory capacity in NAFLD patients [13].

In the current cross-sectional study, we sought to determine if the anti-inflammatory function of apoB-depleted plasma is impaired in NAFLD. Furthermore, we aimed to find out the relationship of anti-inflammatory function of apoB-depleted plasma with NAFLD markers including liver enzymes and liver stiffness.

## Materials and methods

### Study design and participants

This study was approved by the medical ethics committee of Semnan University of Medical Sciences (IR.SEMUMS.REC.1397.329), and written informed consent was obtained from each participant before their participation. The diagnosis of NAFLD was based on abdominal ultrasonography. The subjects were excluded if they had a history of alcohol abuse (30 g/d), diabetes, viral hepatitis, autoimmune liver disease, hemochromatosis, Wilson’s disease. None of the patients were taking medication that has been reported to induce hepatitis. We also excluded the subjects who took antioxidants like vitamin supplements and anti-inflammatory drugs.

### Ultrasonography and elastography

Ultrasound assessment was performed using an Accuvix XQ ultrasound unit (Medison, South Korea) equipped with a 3–7 MHz curved array and a 5–12 MHz linear array transducer for the evaluation of liver, abdominal fat, and carotid arteries as previously described [14]. This technique provides specificity of 100% and sensitivity of 91.7%. Ultrasonographic scores were determined from captured images for vascular blurring (score 0 to 1), hepatorenal echo contrast and/or liver brightness (score 0 to 3), and deep attenuation (score 0 to 2). The diagnosis of NAFLD required a total score of at least 2 [14, 15].

Visceral Adipose Tissue thickness (VAT) corresponds to the distance between the anterior wall of the aorta and the internal surface of the rectus abdominis muscle perpendicular to the aorta. Ultrasonographic measurements have been shown to have strong correlations with the visceral fat area measured by computed tomography [14].

Carotid intima-media thickness (cIMT) was assessed as the distance from the lumen-intima interface to the media-adventitia interface, measured at its thickest point on the distal wall of the common carotid arteries, 1.5–2 cm proximal to the carotid bulb. The average of right and left sides was used for cIMT analysis [14].

Liver stiffness (LS) measurement was performed by transient elastography using the FibroScan 502 machine (EchoSense, Paris, France, 5MHz). According to the manufacturer’s guidelines, the M probe was used for the subjects with a thoracic perimeter less than 110 cm and the XL probe for 110 cm and above. At least 10 measurements were done for each patient and the median value was recorded. When the inter-quartile range (IQR) was > 30% of the median reading the values were considered valid [14].

### Anthropometric and laboratory evaluation

Anthropometric parameters were measured in accordance with the standardized protocols. The body mass index (BMI) was calculated as body weight in kilograms divided by the square of height in meters (kg/m^2^). Waist circumference (WC) was measured midway between the lowest rib and the iliac crest. Blood pressure was measured after 5 min rest at the left arm in a sitting position using an automatic manometer. The metabolic syndrome (MS) was defined according to the revised NCEP-ATP III criteria. According to the NCEP ATP III definition, three or more of the following five criteria are required for categorization of subjects with MS: WC > 102 cm for men, blood pressure > 130/85 mmHg or use of antihypertensive medication, fasting TG level > 150 mg/dl, fasting HDL-C level less than 40 mg/dl for men and fasting blood sugar > 100 mg/dl [16].

Fasting blood samples were obtained from the participants following overnight fasting. Serum and plasma were frozen in aliquots at −80°C for subsequent analysis. Fasting blood glucose (FBG), serum total cholesterol (TC), triglycerides (TG), high-density lipoprotein cholesterol (HDL-C), low-density lipoprotein cholesterol (LDL-C), and levels of alanine aminotransferase (ALT), aspartate aminotransferase (AST), gamma-glutamyl transferase (GGT) were measured by automated enzymatic methods using commercial kits (Pars Azmoon, Iran).

### Cell culture and gene expression analysis

The anti-inflammatory capacity of apoB-depleted plasma was assessed using an in vitro cell system following a recently described procedure [17]. Before analysis, apolipoprotein (apo) B depleted plasma was prepared as previously described. Briefly, 40 μL 20% polyethylene glycol (Merck, Germany) in Glycine was added to 200 μL plasma, mixed, and incubated on ice for 30-minute. Then the mixture was centrifuged at 2200 g for 30 minutes, the HDL-containing supernatant was collected, kept on ice, and used for the measurement of HDL anti-inflammatory function. Human umbilical vein endothelial cells (HUVECs) were isolated from human umbilical cord veins using 0.2% collagenase I (BIO-IDEA, Iran) as described before [18, 19]. All clinical investigation has been conducted according to the principles expressed in the Declaration of Helsinki. Semnan University of Medical Sciences approved the protocols for isolation of HUVECs. Informed written consents have been obtained from the parents. HUVECs were cultured in DMEM supplemented with 10% fetal bovine serum in a humid CO_2_ incubator at 37°C. Early passage HUVECs were seeded at 7 × 10^5^ cells per well in a 6-well plate and allowed to attach for 48 h. Then the cells were incubated with a serum-free medium for 5 h before apoB-depleted plasma was added. After incubation with either apoB-depleted plasma or an equal volume of precipitation reagent in PBS for 1 h, 10-ng/mL tumor necrosis factor-α (TNF-α) (R&D systems) was added for another 5 h to stimulate inflammation. After cell stimulation, total RNA was isolated using the RNA purification kit (GeneAll ® Hybrid-RTM) according to the manufacturer’s instructions. RNA quantification was performed using the Nanodrop (Thermo Nanodrop One). One microgram of total RNA from each sample was used for cDNA synthesis using cDNA synthesis kit (SMOBIO), cDNAs were amplified using a thermal cycler (Eppendorf) in a standard 40-cycle SYBR® green real-time PCR reaction followed by a melt curve analysis to assess amplicon specificity. Gene expression was assessed using the real-time machine (ABI-Step One Plus) with the following primers, VCAM-1: F: GGAGACAGGAGACACAGTA, R: TGGCAGGTATTATTAAGGAGGAT and β-actin: F: GCCTTTGCCGATCCGC, R: GCCGTAGCCGTTGTCG. Quantification of gene expression was normalized to β-actin as a housekeeping gene and analyzed for fold changes in gene expression using the ΔΔCt method.

### Statistical analysis

The Kolmogorov‐Smirnov test was used to test the normal distribution of data. Continuous variables were presented as means ± standard deviation (SD) or medians (interquartile range) and categorical variables were summarized as percentages. Independent-sample t-test or Mann‐Whitney U test was used to analyze differences in continuous variables between the control and NAFLD groups. Associations between the anti-inflammatory capacity of HDL and other variables of interest were tested with the use of Pearson correlation coefficients after appropriate log transformations of the parameters’ values. Binary logistic regression analysis was performed to disclose the independent association of HDL anti-inflammatory data with the HDL anti-inflammatory capacity. Two-sided P values of 0.05 were considered to be significant throughout the manuscript. All of the statistical analyses were performed using the IBM SPSS Statistic 27 and the graphs were prepared using GraphPad Prism 9

## Results

We conducted a cross-sectional study on a total of 83 participants including 42 NAFLD and 41 control subjects who were selected from the Golestan Cohort Study [14]. The Basic clinical and laboratory characteristics of the study groups are described in Table 1. All participants were male, aged 50–81 years old. The control subjects and NAFLD patients had median ages of 59 and 56 respectively. The NAFLD group had higher AST, ALT, GGT, and LS than the control group. Both groups were overweight (mean BMI of >25). But all obesity indices including BMI, WC, WHR, and visceral fat were significantly higher in the NAFLD group compared to the control group. NAFLD patients also had higher FBG compared to the controls. Lipid profiles including Total cholesterol, HDL cholesterol, LDL cholesterol, TG were not significantly different between the groups. The NAFLD and control groups had comparable systolic and diastolic blood pressure. 10 out of 41 control subjects (32.25%) and 16 out of 42 NAFLD patients (38.09%) had hypertension. 17.14% (6 out of 41) of the control subjects and 37.5% of the NAFLD patients (12 out of 44) had metabolic syndrome (MS).

**Table 1:** Baseline characteristics among NAFLD patients and control subjects.

	Control (n = 41)	NAFLD (n = 42)	*P*
Age (years)	59.5 (55.0–62.5)	56.0 (53.0–61.0)	0.167
Systolic blood pressure	134.48 ± 21.73	139.21 ± 23.99	0.352
Diastolic blood pressure	83.12 ± 10.97	85.34 ± 11.39	0.372
BMI (kg/m2)	25.61 ± 2.84	29.49 ± 2.88	<0.001
WC (cm)	95.0 (85.5–99.0)	101.50 (98.0–112.0)	<0.001
WHR	0.95 (0.92–0.98)	0.98 (0.95–1.03)	0.008
TC (mg/dl)	213.22 ± 41.29	211.07 ± 35.77	0.801
LDL-C (mg/dL)	124.34 ± 40.49	128.10 ± 29.47	0.631
HDL-C (mg/dL)	58 (51–67.5)	53 (47–58)	0.027
TG (mg/dL)	126.5 (95.5–196.5)	147 (114–186)	0.330
FBG (mg/dL)	92.71 ± 7.70	99.85 ± 11.88	0.002
Visceral fat	49.59 ± 21.35	67.98 ± 19.01	<0.001
AST (U/L)	20 (16–23.5)	27 (21–33)	0.002
ALT U/L)	17 (13–23)	36.5 (23.0–48.0)	<0.001
GGT (U/L)	21.66 (17.60–31.51)	31.57 (24.33–44.22)	0.001
LS (kPa)	3.75 (3.30–4.35)	6.05 (4.80–8.30)	<0.001
cIMT (mm)	0.75 (0.71–0.85)	0.8 (0.72–0.85)	0.385
MS (%)	14.63	29.26	0.181
Hypertension (%)	32.25	38.09	0.238

Baseline Clinical Characteristics of CAD Subjects and Controls. Independent Student’s t-test or Mann-Whitney U test were used to compare the variables between control and NAFLD groups. Continuous data are expressed as mean ± standard deviation (SD) or median and (interquartile range). Categorical data are presented as percentages. BMI: Body mass index; WC: waist circumference; WHR: waist to hip ratio; TC: Total cholesterol; LDL-C: Low density lipoprotein cholesterol; HDL-C: High density lipoprotein cholesterol; TG: Triglycerides; AST; aspartate amino transferase; ALT: alanine amino transferase; GGT: gamma glutamyl transferase; LS: liver stiffness; cIMT: Carotid intima-media thickness. MS: metabolic syndrome. *P*< 0.05 was considered statistically significant.

**Fig 1:**
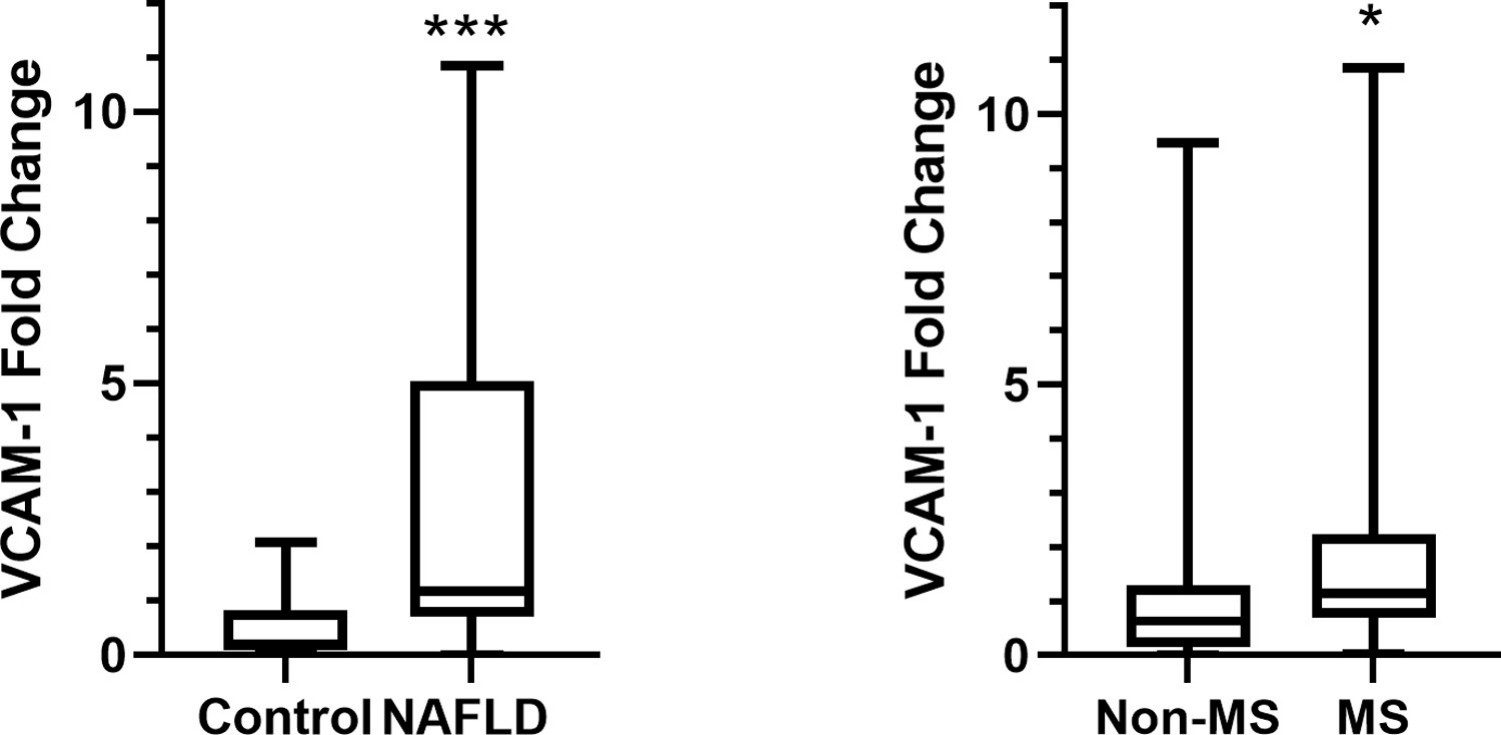
Relative expression of VCAM-1 which indicates anti-inflammatory capacity of HDL. A. The comparison between control and NAFLD groups. B. The comparison between non-MS and MS groups. Differences between the groups were analyzed with Mann-Whitney U; *indicates *P* < 0.05.

The anti-inflammatory capacity of apoB-depleted plasma was impaired in the NAFLD patients [1.18 vs 0.2 fold increase in VCAM-1 mRNA expression; *P* < 0.001] (Fig 1A). When stratified the whole population into MS and non-MS subjects, the anti-inflammatory capacity of apoB-depleted plasma was also different in the MS subjects vs non- MS subjects [1.14 vs 0.63 fold increase in VCAM-1 mRNA expression; *P* = 0.046] (Fig 1B), but was not significantly different between the subjects without hypertension and with hypertension [0.84 vs 0.74 fold increase in VCAM-1 mRNA expression; *P* = 0.984].

Associations between anti-inflammatory capacity of apoB-depleted plasma and the presence of NAFLD were assessed using binary logistic regression. HDL anti-inflammatory capacity was associated with the presence of NAFLD. This association remained significant after adjustment for age and HDL-C. Further adjustment with BMI or visceral fat only marginally attenuated the association of anti-inflammatory capacity of apoB-depleted plasma and NAFLD and it remained significant (Table 2).

**Table 2:** Binary logistic regression analysis for the associations of HDL anti-inflammatory capacity with NAFLD.

Adjusted for	ß	SE	OR (95%CI)	*P*
age, HDL-C	1.512	0.507	4.535 (1.678–12.26)	0.003
age, HDL-C, BMI	1.098	0.464	3 (1.209–7.443)	0.018
age, HDL-C, visceral fat	1.07	0.47	2.916 (1.162–7.321)	0.023

BMI: Body mass index; HDL-C: High density lipoprotein cholesterol. *P*< 0.05 was considered statistically significant.

In the whole population, the anti-inflammatory capacity values (Log FC VCAM-1) of apoB-depleted plasma were strongly correlated with Log LS and Log ALT. HDL anti-inflammatory capacity values of apoB-depleted plasma were also positively correlated with obesity indices including Log visceral fat, BMI, WC, and Log WHR. Moreover, there was a positive correlation between anti-inflammatory capacity values of apoB-depleted plasma and FBG (Table 3).

**Table 3:** Correlation between Log FC VCAM-1 and metabolic and anthropometric parameters.

Parameters	Correlation (r)	*P*
Log cIMT	-0.05	0.656
Log LS	.407**	<0.001
Log age	-0.141	0.205
BMI	.230*	0.039
Log WHR	0.184	0.098
WC	.232*	0.036
TC	0.136	0.221
LDL-C	0.123	0.27
Log HDL-C	-0.049	0.661
Log TG	0.1	0.37
Log AST	0	1
Log ALT	.240*	0.034
Log GGT	0.207	0.061
Log FBG	.223*	0.046

BMI: Body mass index; WC: waist circumference; WHR: waist to hip ratio; TC: Total cholesterol; LDL-C: Low density lipoprotein cholesterol; HDL-C: High density lipoprotein cholesterol; TG: Triglycerides; AST; aspartate amino transferase; ALT: alanine amino transferase; GGT: gamma glutamyl transferase; LS: liver stiffness; cIMT: Carotid artery intima-media thickness. *P* <0.05 was considered statistically significant.

## Discussion

Our findings showed that the inhibition of inflammation-induced expression of adhesion molecules in endothelial cells was significantly impaired in the NAFLD patients. Binary logistic regression analysis demonstrated that the alteration in anti-inflammatory function of apoB-depleted plasma is independent of HDL cholesterol levels. Our results add to the previous studies showed that impaired HDL anti-inflammatory function in other metabolic-related disorders. Dullaart et al. found that anti-inflammatory function of apoB-depleted plasma was impaired in acute MI and their follow-up study showed that HDL anti-inflammatory function was also associated with the new major adverse cardiovascular events in these patients [8]. Jia et al. showed lower anti-inflammatory capacity of apoB-depleted plasma in the subjects who experienced a first cardiovascular event compared to the controls [20]. Annema et al. reported a significant impairment of the anti-inflammatory function of apoB-depleted plasma in the patients with non-ST-segment elevation MI compared to Non-MI participants [3]. Additionally, it has been shown that the anti-inflammatory functionality of apoB-depleted plasma was strongly impaired in type 2 diabetes mellitus [9]. Sang et al. demonstrated an impaired HDL anti-inflammatory capacity in metabolic syndrome subjects [21]. In consistent, our MS-stratified analysis showed an impaired anti-inflammatory capacity of apoB-depleted plasma in metabolic syndrome subjects compared to non-MS subjects.

Our further analysis showed a significant correlation between impaired anti-inflammatory capacity of apoB-depleted plasma and LS as well as ALT serum levels. HDL particle components are vulnerable to modification upon exposure to a range of stimuli including inflammation and oxidative stress which are the main shared risk factors in NAFLD and CVD. Proteomic analysis in HDL particles from NAFLD and normal subjects showed that several HDL proteins were significantly changed in NAFLD [22]. Gene ontology term analysis demonstrated that severity of the liver pathology was associated with alteration in the abundance of HDL-related proteins which had a key role in the anti-thrombotic functions of HDL particles [22]. The relationship between impaired HDL function and severity of NAFLD can be also indirectly supported by another study that showed the association of liver fat content with lower levels of an anti-atherogenic subfraction of HDL [23].

We also observed a significant correlation between impaired anti-inflammatory capacity of apoB-depleted plasma and obesity indices namely BMI, WHR, WC, and visceral fat. Consistently, previous animal and clinical studies showed HDL functions were impaired in ob/ob mice and obese patients [24]. It has been shown that obesity affected the lipid composition of HDL particles, increased acute phase protein serum amyloid a, and altered HDL particle subpopulations. Moreover, HDL of obese subjects showed prooxidative properties [25]. Because of higher obesity indices values in the NAFLD group and a significant relationship between impaired anti-inflammatory capacity of apoB-depleted plasma and obesity indices in the current study, we sought to determine if the association between impaired HDL anti-inflammatory capacity and NAFLD is related to obesity. Our finding showed that the association between impaired anti-inflammatory function of apoB-depleted plasma and NAFLD was attenuated but remained significant after adjustment for the obesity indices. It suggests that the effects of NAFLD on the anti-inflammatory functionality of apoB-depleted plasma cannot be entirely explained by the obesity and involvement of additional NAFLD-specific mechanisms seem likely.

Some limitations to the current study should be noted. The study was cross-sectional which does not allow to draw a conclusion regarding a potential causal relation between the impaired anti-inflammatory function of apoB-depleted plasma and NAFLD. Our sample size was relatively small, and a larger population would be needed to confirm the association of HDL anti-inflammatory function with NAFLD. Our study included only male participants, because previous studies showed gender differences in lipid profile and HDL subfractions of different study populations [26, 27]. Although we removed the confounding effect of gender, the results cannot be generalized to the whole population. We used apoB-depleted plasma to measure anti-inflammatory function of HDL. We did not have enough plasma available to isolate HDL. Further studies are needed to compare the anti-inflammatory function of isolated HDL and apoB-depleted plasma in NAFLD.

In conclusion, NAFLD was strongly associated with impaired anti-inflammatory function of apoB-depleted plasma independent of HDL-C, hypertension, and obesity indices. The impairment of the anti-inflammatory function of apoB-depleted plasma was related to obesity and NAFLD markers.

## Supporting information

S1 Data (XLSX)
